# Experiencing Public Spaces in Southern Chile: Analysing the Effects of the Built Environment on Walking Perceptions

**DOI:** 10.3390/ijerph191912577

**Published:** 2022-10-01

**Authors:** Antonio Zumelzu, Mariana Estrada, Marta Moya, Jairo Troppa

**Affiliations:** Instituto de Arquitectura y Urbanismo, Núcleo de Investigación en Riesgos Naturales y Antropogénicos, Universidad Austral de Chile, Valdivia 5091000, Chile

**Keywords:** walking perceptions, urban experience, built environment, urban design, well-being, Latin America

## Abstract

In Latin American cities, the built environment is facing crucial challenges in the 21st century, not only in terms of the redesign of the physical environment, but also how to remodel public spaces as healthier places for walking and social interaction. The objective of this article is to evaluate the effects of the built environment on walking perceptions in a central neighbourhood in the intermediate city of Valdivia, Chile. The methodology integrates quantitative and qualitative methods to explore which elements of the physical built environment ease and hinder walkability. Depthmap software and Simpson’s Diversity Index are used to evaluate connectivity and diversity of land uses at street level. Additionally, the People Following method and 26 walking interviews are conducted using the Natural Go-Along technique to analyse pedestrians’ perceptions about their mobility environment. The results show that the factors that promote walkability mainly include streets with high connectivity values, wide pavements, diversity of greening, and facade characteristics of buildings with architectural heritage causing tranquillity, longing, and happiness. On the contrary, factors that inhibit walkability are related to poor-quality and narrow sidewalks, cars parked on sidewalks, dirty streets, and motorized traffic and vehicular noise causing negative emotions in walking perceptions such as tiredness, anger, disgust, discomfort, and insecurity, with negative effects on the well-being of residents that vary according to age and gender. Finally, recommendations are oriented to improve public spaces in central areas in southern Chile, to address moving towards more liveable and inclusive environments and support well-being through urban design in these types of context.

## 1. Introduction

Urbanization is one of the most relevant problems countries in Latin America are facing, as it is a heavily urbanized region. Chile is considered to be one of the 10 countries in the world most affected by climate change effects and is confronting important challenges in reducing its carbon emissions and improving the quality of life in its cities [1,2]. In this context, walking in cities, where nine out of ten Chileans live [2], has become a very important practice in preserving the health of the population, especially during the COVID-19 pandemic. In various intermediate cities in southern Chile, small-scale connections within urban mobility networks have lost importance. During the last 30 years, Chilean planning has followed a pattern of creating space for motorized transportation, especially highways connecting sparse urban centres, and neglected active transportation modes such as biking or walking [3]. This situation has generated an increase in the need for a greater number of transfers and excessive dependence on the car, generating environments that promote higher traffic, poor walking environments, pollution, and a loss in quality of public spaces [4,5]. In a climate emergency and rapid urbanization context, which requires the drastic reduction of CO_2_ emissions, the urban built environment is encountering significant issues in the 21st century, not only in terms of the redesign of the physical environment, but also how to remodel public spaces as healthier and safer places for walking and social interaction.

Walking has been internationally recognized as the principal and oldest mode of transport which provides benefits for physical and mental health [6,7]. Walking is also increasingly becoming an essential topic in urban planning, in response to public demand and the pursuit of sustainable urban development in cities [8]. This is recognized in Chilean public policy through the National Urban Development Policy, which defines the objective of promoting walking in cities as “a healthy and sustainable mode of transport” [9]. However, in Chile, people often face several barriers in public spaces while walking, with negative impacts on their mental and physical health [4,10]. Data from the latest mobility survey in Chilean cities [11] show that although intermediate cities register high modal quotas for walking, these have decreased in cities in southern Chile from 33.5% to 23.1% between 2002 and 2013. Similarly, a Chilean Chamber of Construction report notes that 32% of the sidewalks in the main cities of Chile are in a poor condition [12], evidencing the low maintenance of pedestrian spaces [10]. In lower-income neighbourhoods, sidewalks do not even exist or are not paved, and are of poor quality, without trees [13]. These design problems are accentuated by behaviours such as the use of sidewalks for the parking of cars, or their obstruction by means of light poles or traffic signals, which have exacerbated the problem even more during the pandemic period [13,14].

This requires planning and implementing more walkable routes, as well as the development of tools that facilitate the urban design of friendlier and safer public spaces, including sidewalks and intersections, and promote people’s walking in urban areas. To encourage more sustainable mobility in these contexts, it is necessary to advance the development of new tools and methodologies that require support for the urban design of streets and public spaces with greater pedestrian orientation, as a crucial axis for directing the positive transformation of these cities towards a more sustainable future, especially to condition public spaces for safe and healthy mobility in the pandemic and post-pandemic periods. Considering the above, this study formulates the following research question: what elements of the built environment may affect the walking perceptions of residents, to improve current environments and support well-being in intermediate cities?

The objective of this article is to evaluate the effects of the built environment on walkability in a central neighbourhood in the intermediate city of Valdivia, Chile, based on the perceptions of walkers. This research focuses on intermediate cities in southern Chile, not only because they have undergone accelerated spatial transformations that have affected the quality of the physical infrastructure of their public spaces [15], but also because they have suddenly increased their population as a result of the COVID-19 pandemic and the social demands—the social outburst—that have emerged since October 2019 in Chile in response to social inequality prevalent in the country, presenting complex challenges for urbanization and the quality of life in cities. On the other hand, southern cities in Chile are associated with better well-being outcomes than other cities in the country, due to various factors, including the type of environment in which the residents live [12,16,17]. In addition, the city of Valdivia has been recognized at the Latin American level as having great potential, as well as attracting interest from governments for promoting sustainable urban development [18,19]. Specifically, the central neighbourhood, Barrios Bajos, is considered in this case study. The district has a prominent role in the city’s history. Its pericentral location, and therefore the potential linked to its centrality in the city, have promoted new urban dynamics, which as a whole have induced a recent urban transformation process in the area.

This article is structured in three parts. First, the literature on the built environment and its relationship with walkability is reviewed. Subsequently, an empirical analysis is carried out integrating qualitative and quantitative methods to explore which elements of the built environment ease and hinder walkability. To analyse the morphological properties of the built environment that influence walkability, two spatial analysis software programs (Depthmap and ArcGIS) are utilized. To assess the distance a person walks between their points of interest and the choice of routes, the People Following method, derived from space syntax theory, is employed [20,21]. The Walking Interview method, through the Natural Go-Along technique [22,23], is applied to obtain records of the self-declared perceptions of pedestrians about their urban mobility environment. Finally, the results are used to formulate recommendations and suggestions for the urban redesign of public spaces, to address moving towards more liveable environments, and to support well-being in the central neighbourhoods of intermediate cities in Latin America.

### 1.1. Built Environment and Walkability

Walkability is a wide concept; it has been defined in many ways, as proven by the extensive literature available [24]. Many international definitions have focused on the role of mobility experience. In this sense, walkability has been defined as an everyday activity and an expression of rights, with an important impact on equity and health [25,26]. For example, Krambeck considers the term of walkability in its most basic sense: “the safety, security, economy and convenience of traveling by foot” [27]. Bharucha defines it similarly as “the extent to which walking is readily available as a safe, connected, accessible and pleasant mode of transport” [28]. While Kelly et al. define walking as a “mean of experiencing and interacting with the local environment and wider society in a way not possible when using particularly motor transport” [29]. However, the international literature has also evidenced that one of the key aspects in promoting walking is the built environment. In this sense, walkability has been defined by “the quality of which the built environment enables the mobility of pedestrians” [4,8]. Southworth also highlights the role of built environment in its definition, in which it has to provide “comfort and safety for pedestrians, connecting people with varied destinations with a reasonable amount of time and effort, and offering interest in the journey throughout the network” [30], while Lo states that to understand the concept of walkability, it is important to consider not only the built environment but also how pedestrians are defined and the discourses that shape the development of pedestrian space. He defines walkability as an exploratory concept, “highlighting the need to analyse the different facets of walking and its implications for pedestrian quality of service” [24]. Along this line, authors agree that walkability is a multidisciplinary activity that requires multiple metrics to understand it, not only the built environment, but also different facets emphasising personal space and those which advocate safety, comfort and other issues, and that could vary according to different social and geographical contexts.

Many international studies have sought to identify the variables of the built environment that are related to promoting or inhibiting walking [31,32,33]. These vary according to different scales [25]. As international research has shown on larger scales, most recent evidence suggests that built environmental variables such as population density, connectivity and mixed uses positively influence people’s willingness to walk [34,35,36,37].

Density and a mix of land uses are often seen as fundamental factors for promoting walking [29], since they shorten the distances between people and the places that need to be accessed. Traditionally, many investigations have indicated that residents in compact urban areas with good access to local amenities are more likely to walk than those living in sprawling ones. Residents living in urban locations with well-provided services and facilities and mixed land uses are more likely to walk because of the increased access to destinations through walking [38,39,40]. However, different conclusions have also been noted. For example, Hong and Chen find in the United States that density has a positive association with walking, but it can also negatively affect people’s perceptions towards crime safety. They demonstrate that density could be associated with heterogeneous and potentially deviant behaviours. Specifically, they argue that residents living in neighbourhoods with good access to facilities by walking tend to perceive their neighbourhoods as safer, while density has the opposite impact [41]. Similarly, Zumelzu et al. observe in an examination of cities in southern Latin America that walking decreases in places with a high dwelling density due to the expansion of single-family homes within the plot, producing negative effects on public spaces. Elements such as blind walls or blank frontages along the street, the absence of front gardens, a low distance between facade fronts, and a decrease in the width of sidewalks negatively affect the choice of routes and consequently walking. This is because the perception of crime increases on these routes, mainly in spaces with low visibility. According to their analysis, four conditions of urban form are associated with promoting greater walkability in these contexts: housing density, the diversity of uses, the quality of housing typology, and the presence of green areas [37]. However, Pivo and Fisher state that a development with an appropriate density, and an efficient mixed use of land, could encourage a high walkability, increasing the possibilities for social interaction in the urban space. They demonstrate in the United States that the presence of a diversity of uses contributes to creating a sense of vibrancy and liveliness in an urban space, boosting the attractiveness of an urban area, raising not only its economically evaluation, but also maximizing the value of property rates [42].

Another factor of the built environment that encourages walkability is connectivity. Generally, connectivity refers to the degree to which local environments offer points of connection and contact at a variety of scales and for multiple purposes [43]. It is also defined as a morphology property of urban space, by the number of street intersections and lengths of urban blocks as determinants for people to take shorter and more direct paths to their destinations [44,45,46]. Unlike land uses, high connectivity is found when streets are laid out in a grid pattern and there are few barriers to direct movements between origins and destinations. When connectivity is high, route distance is similar to a straight-line distance and easier to walk. By contrast, low connectivity is found in the layout of suburbs, characterized by a low density of street intersections, long urban block sizes and few route choices [47]. Many studies have mentioned the importance of smaller urban blocks—between 60 and 90 metres long—based on their apparent benefits for walking [48,49]. Leon Krier, for example, argues that urban blocks in small cities generate greater diversity and complexity in the urban landscape. According to Krier, urban blocks should be as small in length and width as is typologically feasible. These should form as many well-defined streets and squares as possible in the form of a multidirectional horizontal pattern of urban spaces [50].

Reaffirming the above, in the 1960s Jane Jacobs suggested that most urban blocks should be short; that is, streets and corner-turning opportunities should be frequent [51]. According to her work in the United States, shorter urban blocks would allow more encounters and greater social interactions between people. Similarly, in the United States in the 1990s, Allan Jacobs posited that the frequency of street crossings—or a great number of street intersections—contributes to the diverse pedestrian qualities of a street: “streets with one entrance for every 90 m are easy to find, and some of the best streets are close to that indicator, but there are more entrances on the busier streets” [52]. Most recent international research studies agree that indicators of “good” functioning imply that blocks of a length between 60 and 70 metres are well meshed and optimal for pedestrians, and those up to 100 metres well meshed and very convenient for pedestrians, while those over 200 metres are very inconvenient for promoting pedestrian mobility [53,54,55,56].

In more recent years, many studies have focused on the role of the “micro” components of the built environment, such as the width of sidewalks, the presence of trees or the existence of benches, and other elements that could encourage or inhibit walking [13,57,58,59]. For example, Sevtsuk et al. [54] point out that shorter blocks are not necessarily better for pedestrian traffic, but include other conditions such as the presence of trees in the street, wide sidewalk width, street fronts with high porosity as well as with parking lots, blind walls, or fronts with glass facades, and whether there is sufficient connectivity between the streets and a low volume of traffic. This has been reaffirmed by other research that has identified the infrastructure of public space over block size, which has important effects on people’s perceptions, especially during the pandemic period [60].

### 1.2. The Built Environment and Its Effects on Walking Perceptions

Physical features of street design could also play an important role in walking and individuals’ perception of the activity, and they can influence a specific feeling about the environment as they walk through it [61,62,63]. In this sense, relevant quantitative assessment tools have been development to explore the effects of physical environment on walking perceptions. For example, Krambeck developed a Global Walkability Index (GWI) to rank cities within a country and across the world based on the safety, security, and convenience of their pedestrian environment. It includes universally applicable variables, which has been applied in different Chinese, US, and Indian cities [64]. Based on this tool, The CAI-Asia developed a Walkability Index and survey for Indian cities that looks at twelve parameters to assess walkability. This survey can be used to improve design aspects of streets, by enabling the surveyors to have a scoring system for their city or neighborhoods [65]. Similar tools have been developed in the USA and applied in different cities worldwide such as the Neighborhood Environment Walkability Scale (NEWS) [66], and their versions NEWS-A (simplified version) and NEWS-Y (Youth version) [67,68].

However, different authors argue that individual perception—such as a sense of comfort, safety perceptions, and level of interest—can only be measured subjectively because they reflect how individuals react to a place and how a person assesses the conditions there, considering only their own attitudes and preferences [69,70,71]. In this sense, recent studies have shown the relevance of using new visualization techniques (virtual reality technology) to evaluate how groups of people perceive walkability differently at neighborhood and street levels, especially during pandemic crises [36,62].

Unlike other international walkability studies such as in Europe and the USA, in Latin America, qualitative applications have prevailed over quantitative ones. Qualitative methods such as pedestrian-focused surveys, walking interviews, or following traces have been preferred by authors in Latin America, to the detriment of data-based models such as GIS, Walkscore indexes and others. However, there are similarities with the scale analysis and conceptual approach of public space analyses, such as those conducted by Viktor Mehta [72] or Emily Talen [73] in the USA.

From a methodological perspective, qualitative methods such as walking interviews or the Natural Go-Along technique have the advantage of generating spatially sensitive insights from being carried out in situ, walking the streets and being in motion, which gives them greater ecological validity, given the environmental (temperature, noise, smells, and other aspects), emotional (happiness, anger, fear, sadness, disgust, surprise, trust, or anxiety) and social variables (the presence of people, etc.) in the streets that affect the walking experience [13,22,23,69]. In these contexts, urban studies about the perception of the physical environments of pedestrian mobility are of great interest for both architecture and urban street design, since they yield results about the preferences of pedestrians in relation to their physical urban environment.

In terms of walking perceptions, research to date has shown important associations between gender differences and physical design of streets and neighbourhoods. For example, Pollard and Wagnild made a systematic review to assess the current evidence on gender differences in walking in high income countries. Their results reveal that women report a higher prevalence than men of walking for leisure, for exercise, and for fun when all ages are considered together. In fact, the study shows that more women than men walk for leisure at young adult ages, in which child-care plays an important role in the relatively high levels of walking, especially in areas with great access to greening and public squares [74]. On the other hand, Risová and Madajová demonstrate in a study implemented in Slovakia, that there are important gender differences in the perception of being safe in public spaces in central neighborhoods. Specifically, they evidence that fear-related spaces were more dispersed for boys than for girls, who perceived risky areas were more compact and spatially concentrated, especially related with certain types of use of land (pubs and urban greenery in particular) at night. In risky areas, women prefer to walk with another person or at least with a dog, as they are more sensitive to the gross behaviour of strangers and to signs of disorder and rubbish according to this study [75]. Similarly, Hidayati et al. investigated the interplay that shapes perception of safety in relation to the spatial configuration of the built environment and its socio-cultural construct in two cities in Malaysia to understand how this interrelation affects gendered mobility. In their study, they found a large proportion of women ascribed to negative perceptions of safety as compared to men, especially related to built environmental cues such as visibility of street frontages, the image of the place and the presence or absence of types of land uses [76].

In Latin America, recent evidence has shown that the social aspects of the neighbourhood influenced by their physical design, such as the fear of crime and gender differences, represent the perceptions more experienced by people in terms of a neighbourhood’s walkability, especially in low-income areas [77,78,79,80,81]. For example, Herrmann-Lunecke is one of the greatest exponents in Latin America of studies of urban space and walkability. In her study, based on walking interviews as a method applied to central neighbourhoods in Santiago de Chile, she demonstrates that the experience of walking is strongly determined by “micro” elements of the built environment: not only by the presence of sidewalks, but also by their characteristics (distinguishing width, cleanliness, surface texture, and quality) being determining factors in enabling greater safety, comfort and, in many cases, positive emotional well-being [13,33,82]. To improve the subjective well-being of people, the design of public space should consider the interests of users so that it can be safe and inclusive. A safe sidewalk in a pedestrian-friendly environment could increase the safety perception, comfort, and efficient displacement of all its users [49,61]. In addition to the sidewalks, other elements such as the characteristics of building facades, as well as the presence of trees, pedestrian crossings, vehicular traffic, and noise also stand out as determining factors.

Walking is a free activity and its benefits at the neighbourhood level are associated not only with the scope of equity and the increase of social capital at the community level, but also with personal well-being related to the longevity of good mental and physical health [83,84,85,86,87]. Promoting walkability is an essential part of urban sustainability, in which the quality of the built environment and its physical elements play a fundamental role in meeting its challenges. However, investigations in Latin America, specifically in Chile, are still very incipient and more research is required on which physical–spatial factors of the built environment promote and/or inhibit walking in both neighbourhoods and urban areas.

## 2. Materials and Methods

### 2.1. Case Study

**Figure 1:**
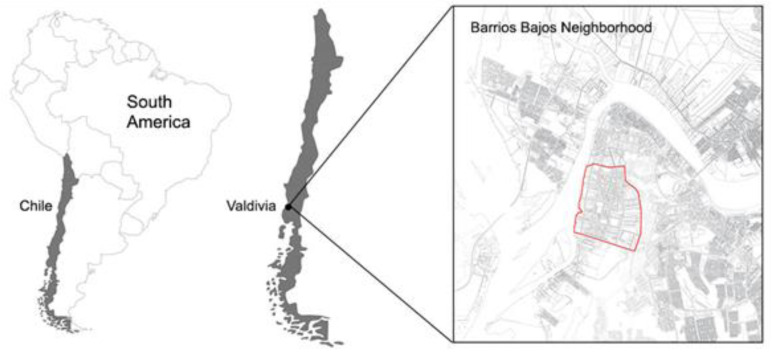
Barrios Bajos Neighbourhood location in the city of Valdivia. Source: Authors.

The Barrios Bajos neighbourhood (Figure 1) has a prominent role in the city’s history. It represents one of the first extensions of the foundational area, to the south, following the Camino Real, today the Yungay–General Lagos axis. When it was first built, it was a sector of farms and grazing, delimited during the second half of the 18th century by a system of urban fortifications. Very soon, the city’s growth marked the differences within this neighbourhood. On the one hand, the particular and unique development of General Lagos Street, linked to the river and characterized by production facilities and German immigration architecture, was declared a heritage protection zone for its value. On the other, it was a plot with a working-class character in its beginnings which was settled on the lowest soils of the city, avoiding the most serious floods in the urban history of Valdivia [88].

In recent years, the Barrios Bajos neighbourhood has been characterized by certain levels of obsolescence, including the presence of deteriorating heritage, low public investment, little private dynamism, and the presence of sectors with high urban and housing vulnerability. However, its location, linked to its centrality in the city, has promoted new dynamics that have induced a recent transformation process in the sector. This transformation is evidenced by the presence of educational services such as universities, primary schools, and kindergartens, in addition to other services associated with education such as lodging for students, restaurants and laundries [89].

### 2.2. Methods

This article explores which elements of the physical built environment ease or hinder walkability. Table 1 summarizes the methodological structure for evaluating walkability. The methodology has been applied through fieldwork and quantitative methods such as the employment of analytical instruments, as well as qualitative ones such as observations and surveys (Table 1).

To assess the distance a person walks between their points of interest and the choice of routes, the People Following method [20] is applied. By tracking people, the average distance people walk between their points of interest and their route choice is measured. To track routes, a radius between 400 m and 800 m is defined. When using this technique, it is recommended to follow about 25–50 people, per observation day. The observation days are for two days, Wednesday morning and Friday afternoon. Moreover, it is important to set user categories. In the case of this study, the categories are young people, adults and the elderly. To apply the method, first the observer needs to stand on the chosen point of distribution. Then, the observer has to pick up a person and follow him/her at a prudent distance to prevent him/her from noticing the presence of the observer. The observer must trace the route taken by the person on a printed map, until the person reaches the destination or when the person reaches the radius of study. As result, a graph is obtained that shows all the routes to find movement patterns [20]. In this study, 40 people are followed. The distribution points selected are the main streets within a radius of 400 m. These streets are General Lagos, Perez Rosales, Lord Cochrane, and Baquedano. This field observation includes determining the quality of the built environment as a factor that may affect path choice with better or worse spatial quality.

Two morphological properties of the built environment are evaluated: the connectivity of streets and the diversity of land uses. Connectivity is measured by the transect method, counting intersection numbers per area unit using Depthmap software analysis derived from space syntax theory [21]. This method helps to measure the density of street centre lines and the number of intersections per unit of area. Land uses and mixture are calculated through the ArcGIS software, using the Shapefile format and polygon geometry (National Institute of Statistics, INE). The information obtained has been updated to 2020 through fieldwork analysis. The diversity of land uses is assessed using Simpson’s diversity index, a measure commonly used in ecology and adapted by Talen [90], to determine diversity in communities and quantify the diversity of a residential habitat, considering the number of units present, as well as the abundance of each unit in the territory of southern Chile. It is given by:

D = 1 Σ n(n − 1), N(N − 1)(1)

where:

- D = diversity;
- n = number of individuals in each unit;
- N = total number of individuals in all units.

Values of D range between 0 and 1. High scores (close to 1) indicate high diversity. Low scores (close to 0) indicate low diversity.

Finally, to analyse the perceptions of pedestrians in regard to their mobility environment, walking interviews through the Natural Go-Along technique [22] are employed to obtain records of the self-declared perceptions of pedestrians of their urban environment while walking. In the application of this method, the researcher walks with the interviewees as they go about their daily routines, asking them questions along the way [22,23,69]. According to Kusenbach [22], the go-along method is helpful for capturing hidden or unnoticed habitual relations with places and the environment, since it tends to highlight environmental perception, spatial practices, biographies, and social realms, among other aspects, in the data gathered. To carry out the interviews, ethical approval was obtained by the Ethics and Bioethics Committee of the Universidad Austral de Chile and informed consent was shown to the participants.

Twenty-six walking interviews were conducted using the Natural Go-Along method, including 13 interviewees who were women. Participants were recruited with the help of the neighbourhood associations. All participants had to have been residents of the neighbourhood for at least two years and within an age range between 18 and 80 years old. This made it possible to analyse differences in perceptions according to age and gender. A “type” route was identified; that is, a route that a large part of the inhabitants of the neighbourhood used daily, with the results of the People Following method.

The distance walked for each interview was within a radius of 400 m and 800 m (between 10 and 20 min walking), a measure internationally associated with the pedestrian walk threshold [13]. A walking interview was conducted with each participant to record the residents’ self-reported perceptions of their urban environment while walking. Each participant was asked to freely narrate their walking experience and to point out which elements facilitated and which inhibited their walk, expressing their emotions while walking. Each conversation was recorded with a smartphone, and the Strava app (GPS) was used to georeference the walked routes and to verify the location of each record. Also, pictures were taken during the walking interviews, according to the elements that interviewers highlighted while walking. The walking interviews were carried out on weekdays, between the months of November and December 2021, and March 2022, during the spring and summer periods. The interviews were coded and processed in the qualitative analysis software Atlas.ti, based on the open coding procedure described in the Grounded Theory method [91]. Two researchers were involved in this task.

Finally, the participants produced the data through the action of walking and talking, but were not involved in the analysis of the resultant data and maps.

## 3. Results

Within the area under study in the Barrios Bajos neighbourhood, the pattern of the street system is characterized, to a great extent, by a rectangular grid showing a high physical permeability in its plot, which allows for ease of movement through the unit. The area has a system of interconnected streets with a pattern that promotes a greater possibility of pedestrian movement.

**Figure 2:**
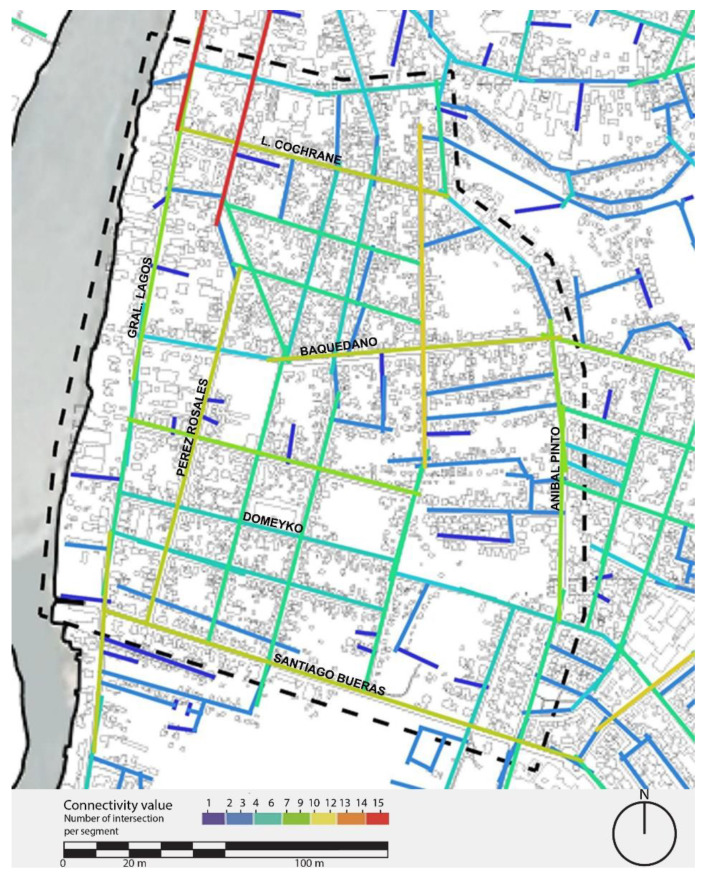
Connectivity map of the Barrios Bajos, Valdivia. Source: Authors.

In the axial connectivity analysis of Figure 2, the main streets, Pérez Rosales to the north and Calle Yungay, stand out as the ones with the highest connectivity in the sector, with a value of 15 (number of connections per segment). These are followed by the main streets within the neighbourhood, such as Clemente Escobar (12), Coronel Santiago Bueras (11), Pérez Rosales to the south (11), Lord Cochrane (10), and Baquedano (10). By contrast, interior passages and streets maintain low levels of connectivity. Barrios Bajos has an average connectivity value of 4.8, a medium average. The reason is mainly that it is a neighbourhood with low internal connectivity, due to the high presence of internal streets and alleys and the great connectivity of its perimeter streets, such as Lord Cochrane, Pérez Rosales Norte, Coronel Santiago Bueras, and General Lagos streets.

**Figure 3:**
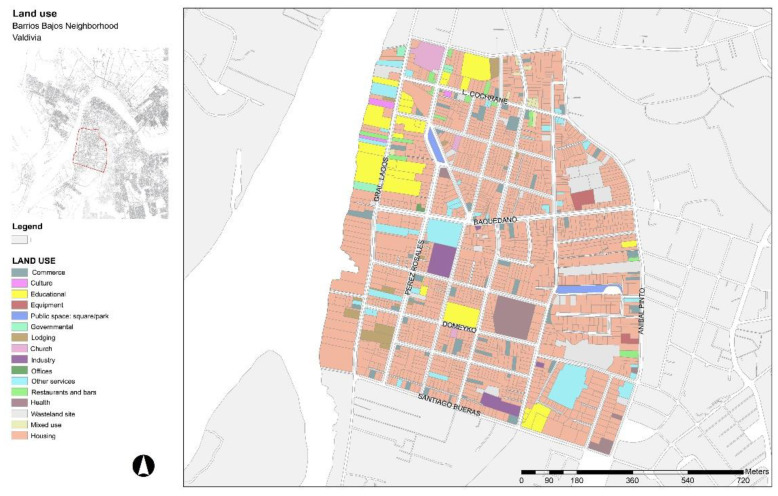
Land uses map of the Barrios Bajos, Valdivia. Source: Authors.

Regarding the diversity land use analysis, Figure 3 and Table 2 show the streets with the highest non-residential land uses and the highest diversity index. Within the neighbourhood, three streets are highlighted with a high value of diversity of land uses. The street with the highest diversity value corresponds to Cochrane Street. This street has a commercial character mainly aimed at university students, of which approximately 39.84% corresponds to non-residential uses, in which services and businesses classified as “daily supply” predominate. The mixed building use corresponds to a low 2.21%, with shared use between housing and services such as restaurants and local trades, local commerce, and a kindergarten. The diversity index in this street is high, 0.66 according to the Simpson’s diversity index. The non-residential uses mainly involve reused homes (75%) while a much smaller percentage includes buildings designed for a commercial purpose (25%).

On the other side, General Lagos Street also shows high values of land use diversity. This street has an important educational vocation. The appearance of primary and secondary schools has generated the appearance of various local shops and services along the street, transforming this section into an important node for cultural, educational, and recreational activities with a strong amount of pedestrian and vehicular activity. The street has a high percentage of non-residential uses (42.7%) destined mainly for educational, cultural, and sport facilities, and local commerce. The diversity of uses of this street is high (0.69), according to Simpson’s diversity index.

Finally, Pérez Rosales Street has a high pedestrian movement and vehicular load, due to its moderate value of diversity of uses (0.44) according to Simpson’s diversity index. This street includes a central square at its centre, Pastene Square, which is the most important public space in the neighbourhood in terms of community recreation. It is a public space with sports equipment, green areas, furniture, and children’s games. The street has a mainly residential character, with 23.47% of buildings with non-residential uses in relation to the total number, destined for a health centre, a chapel, a café, and a retail-type business (Table 2).

### 3.1. Assessing Choices of Route: People Following

The People Following method is applied to identify the most used pedestrian routes within the study polygon as well as the streets with the highest pedestrian agglomeration, evaluate what environmental factors influence the choice of a pedestrian route, and determine the distance that a person walks between their points of interest.

**Figure 4:**
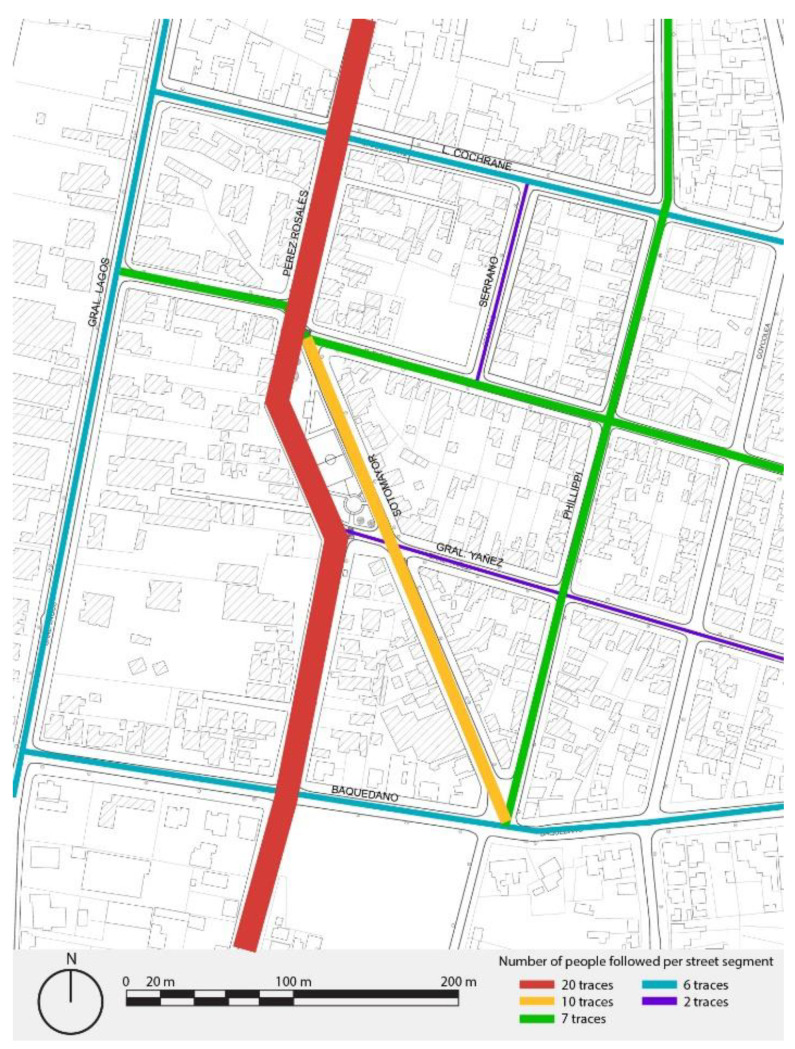
Pedestrian movement pattern map of Barrios Bajos, Valdivia. Source: Authors.

As a result of the application, a high pedestrian flow can be seen on Pérez Rosales Street, followed by General Lagos Street. The busiest areas are related to places in the public space, for example, recreational, such as Pastene Square, and educational, such as colleges and universities. Moreover, a number of highly used paths are observed, such as the case of the west path of Pérez Rosales, which was recently improved and widened, while the east path shows greater use in the section where it widens from Riquelme to the north (see Figure 4).

When relating the results of connectivity, the diversity of land use analysis, and the movement patterns resulting from the People Following technique, a correlation is observed between the sections with the greatest connectivity, such as Pérez Rosales and Sotomayor streets, with the route choice of pedestrians who circulate along the area. The streets with a higher diversity of uses, such as General Lagos, Pérez Rosales, and Cochrane, show a major movement pattern of pedestrians within the neighbourhood. Certain services and facilities are located in these streets, such as two health centres and Plaza Pastene. On the other hand, when observing the quality of the space, the area has more trees, wide sidewalks, and homogeneity in its textures. These elements provide greater urban vitality to the sector, through the use of the square and the diversity of activities carried out in the public space.

### 3.2. Assessing Pedestrian Perception: Walking Interview—Natural Go-Along Method

**Figure 5:**
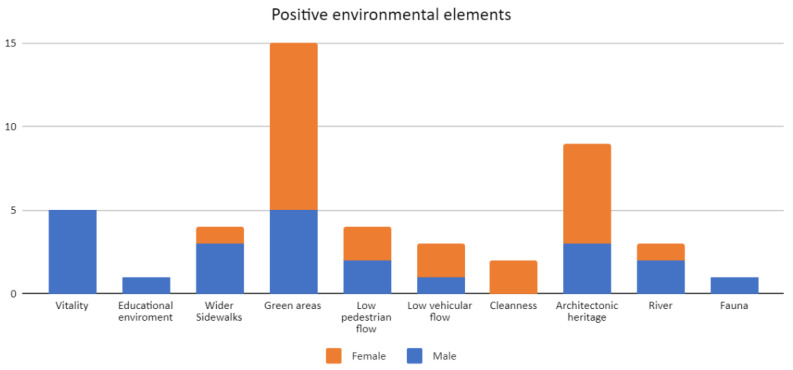
Positive environmental elements mentioned during walking interviews in Barrios Bajos, Valdivia. Source: Authors.

The results showed that most participants (69%) were between 18 and 30 years old. From this range, nine were women and nine men. Two participants, one woman and one man, were elderly. The rest of participants were between 31 and 50 years old, from which three were women and three were men. The pedestrian perception analysis presented three relevant results. First, the positive aspects of the urban environment mentioned included the vitality, the educational environment, the wide sidewalks, the green areas, the low vehicular flow, the cleanliness, the architectural heritage, the river, and the fauna. Of these elements, the green areas, noted on 15 occasions, and the architectural heritage, referenced nine times, were prominent. On the other hand, it was highlighted that the perceptions of the positive aspects varied according to the gender of the interviewees. Most women interviewed emphasized green areas and architectural heritage as positive aspects, mainly in Sotomayor and some segments in General Lagos Street and Pastene Square. In the case of men, vitality and green areas stood out, as shown in Figure 5. In the words of a 25-year-old woman (General Lagos Street):

*“Seeing the colors (of the facades and trees), the street is small, cozy, I don’t know how to say it, well, and since there are plenty of people it feels like a street that has a lot of life. It is difficult to describe it, but it is like that, that the city is alive, that the street is alive, there are relationships, things happen”*.

General Lagos and Somotayor streets were perceived positively by most of the interviewees. For example, the positive emotions related to architectural heritage were not only associated with the characteristics of facades and façade heights, but also with a sense of belonging associated with an attachment to the place, because a relative lived there or the interviewee used to live there during his childhood, which was sometimes mentioned in expressions such as the “old street”. Mainly, these perception differences were strongly marked by the age of the interviewees. In the words of a 51-year old woman and a 23-year-old woman, and of a 21-year-old man (General Lagos Street):

*“I feel nostalgic walking down this street because I’ve spent my whole life here, it’s still a super old street, my whole family lived here”*.

Words of a 51 year-old woman.

*“I have lived a long time in this sector[...]. I like the fact that they are not large buildings, that there are no skyscrapers and that they are low-rise buildings. So, I like the style and it makes me happy to walk by this street”*.

Words of a 23 year-old woman.

*“I love the colors of the facades, the street is like little, like cozy[...], it feels like a street that has a lot of life”*.

Words of a 21 year-old man.

The positive emotions related with green areas were associated not only to the quantity of greening but also to the diversity of vegetation, plants, and their characteristics on the streets and squares. There were no gender or age differences observed in the perceptions. In the words of a 41 year-old woman and a 19- and a 30-year old man (Pastene Square, in Sotomayor street):

*“I like to walk down this street because it is quiet, there are many trees in some parts”*

Words of a 41 year-old woman.

*“I like to walk around here because there is a diversity of trees and plants, and it is generally quiet”*.

Words of a 30 year-old man.

*“I don’t know, there is a lot of vegetation like here, there are many types of plants and things like that [...]. Group of trees, shadows and the leaves of the trees, it gives me a feeling of tranquility walking here, of peace”*

Words of a 19 year-old man.

Second, the negative aspects of the urban environment included flooding, noise, garbage, crowding, high vehicular traffic, vehicles parked on sidewalks, and sidewalks in poor condition, especially in Cochrane and Pérez Rosales streets. Of these elements, the high levels of vehicular traffic mentioned on seven occasions, the vehicles parked on the sidewalk referenced five times, and the sidewalks in poor condition noted four times stand out. In the words of a 53-year-old woman (Pérez Rosales Street):

*“The garbage [...]. Makes me feel like the street is dirty and not kept up [...]. It generates me like anger, disgust, that is”*.

**Figure 6:**
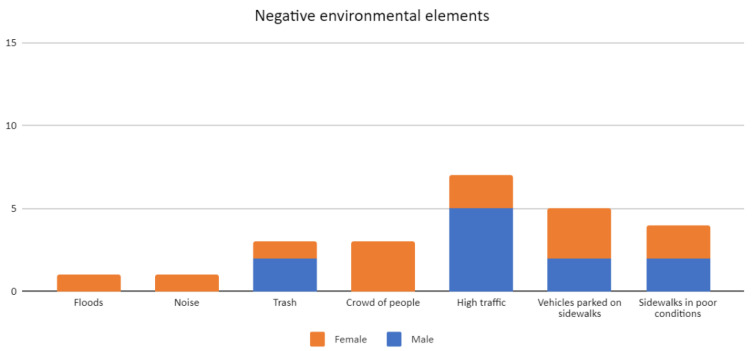
Negative environmental elements mentioned during walking interviews in Barrios Bajos, Valdivia. Source: Authors.

Likewise, it was evident that the perceptions of the negative aspects varied according to the gender of the interviewees. Most of the women interviewed highlighted the crowding of people and the vehicles parked on the sidewalks as negative aspects. In the case of men, the high level of vehicular traffic was conspicuous, as shown in Figure 6. In the words of a 63-year-old man (Cochrane Street):

*“There are times when the street narrows and many people come past and we have to go down the street, go up the sidewalk again. then it is quite dangerous”*.

*“I value this route because there are not so many people and that in reality, I do not like walking through such crowded streets”*.

Words of a 19 year-old woman.

Also, the perceptions of the negative aspects varied according to the age of the interviewees. For example, most young people interviewed positively perceived the high vitality of the streets, as opposed to adults, the majority of whom perceived the crowd of people and high levels of traffic negatively:

*“I like this street because there are a lot of people, there is a lot of traffic anyway, it feels like a street that has a lot of life”*.

Words of a 21 year-old man.

*“What I value most when walking this route is the movement, seeing lots of people, more students, there are lots of people”*.

Words of a 23 year-old man.

**Figure 7:**
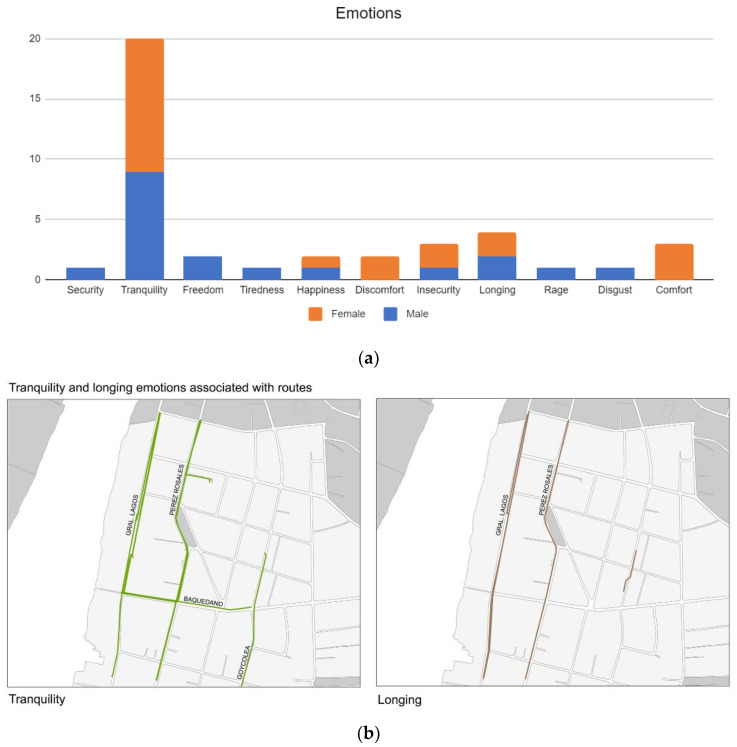
(**a**). Emotions mentioned during walking interviews in Barrios Bajos, Valdivia. Source: Authors. (**b**). Emotions associated with chosen routes in Barrios Bajos, Valdivia. Source: Authors.

Third, people declared various sensations generated by the route they chose, including: security, tranquillity, freedom, tiredness, happiness, discomfort, insecurity, nostalgia, anger, disgust, and comfort. Two of these sensations stood out: tranquillity, noted on 20 occasions, and nostalgia, noted four times, as shown in Figure 7a. Figure 7b, which show the emotions associated with each of the chosen routes.

Emotions of tranquillity and longing were mostly associated with General Lagos, Baquedano, Goycolea, and Perez Rosales (Pastene square) streets. Elements associated with emotions of tranquillity were the abundance and quality of greening of streets, referred to in the diversity of plants, trees and their characteristics. While elements associated with emotions of longing were physical (facades’ characteristics and buildings’ heights) and non-physical components (sense of belonging) of architectural heritage. Tranquillity and longing were perceived without much difference of gender. However, longing associated with non-physical components was demonstrated mostly by adults and the elderly, as opposed to younger interviewees who associated longing with physical components of architectural heritage, showing important differences in terms of the ages of the interviewees. In terms of negative emotions, discomfort and insecurity were perceived mostly by women, especially associated with crowded streets, sidewalks in poor condition, and high levels of traffic and noisy streets.

## 4. Discussion

This study showed how aspects of the built environment affect the walkability of people in the Barrios Bajos neighbourhood. Regarding individuals’ preferences, the survey results demonstrated that people are more likely to take the most connected and shortest way to reach their destination by foot. The quality of the space, as well as streets with trees, wide sidewalks, and homogeneity in street surfaces, are elements related to providing greater urban vitality to the sector, based on the use of the square and the diversity of activities carried out in the public space. These results relate to what has been proposed by various investigations which evidence that streets with high connectivity values, as well as a diversity of land uses and high quality of public spaces, are related to high urban vitality and route distances that are easier to walk [47,48,49]. However, some “micro” components of the built environment could be determinants that affect people’s route preferences.

**Figure 8:**
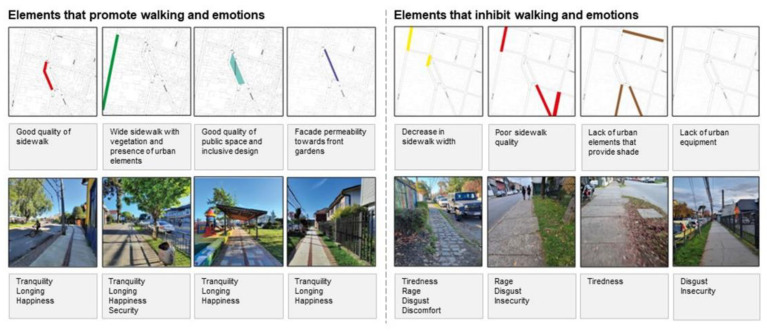
Elements that promote or inhibit walking, based on walkers’ perceptions in Barrios Bajos, Valdivia. Pictures were taken during interviews with participants, according to the elements that interviewers highlighted while walking. Source: Authors.

**Figure 9:**
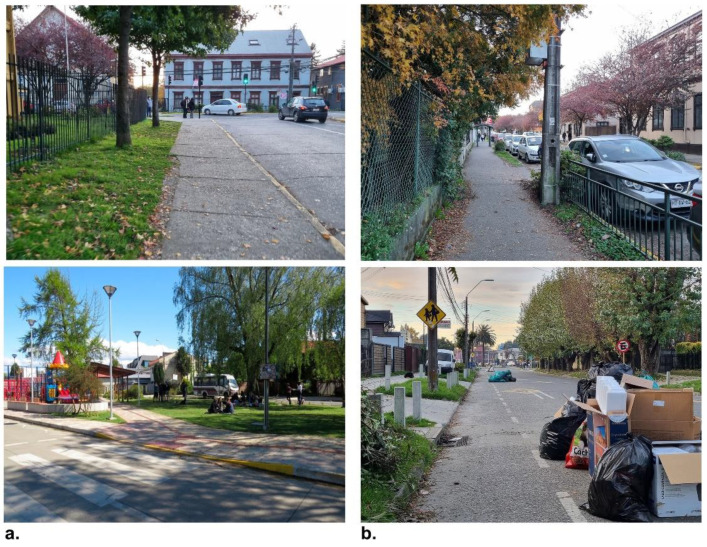
(**a**) Trees on streets, wide sidewalks and architectural heritage on street frontages are the most preferred elements that promote walking based on the perceptions of walkers; (**b**) parked cars on sidewalks, narrow sidewalks and garbage on inner streets are elements that inhibit walking, based on the perceptions of walkers. Pictures were taken during interviews with participants, according to the elements that interviewers highlighted while walking. Source: Authors.

Elements that promote or inhibit walking, based on walkers’ perceptions, are classified in Figure 8. Accordingly, of the identified elements that promote walking, the good condition and quality of the sidewalks stand out, as well as wide sidewalks (with a two-metre width), the provision of urban elements such as roofs, benches, and street furniture, public spaces with inclusive design, green areas, and tree masses and diversity of vegetation, in addition to shade and the permeability of the façade towards front gardens. Likewise, these elements can be associated with emotions such as tranquillity, nostalgia, happiness, and the security of neighbourhood residents (Figure 9a). These coincide with the findings of other authors that evidence that the quality of the infrastructure of public spaces can be one of the determinants that encourage or inhibit walking [13,58,59], as opposed to only morphological properties of the urban fabric such as block sizes, connectivity, or accessibility, which have important effects on people’s perceptions [6,60]. In this sense, the residents of this central neighbourhood highlight the architectural heritage of facades and building height characteristics and the quality and diversity of green elements of streets as the most important elements of the built environment that promote positive emotions such as longing, happiness, or tranquillity while walking (Figure 9a).

On the other hand, elements that inhibit walking, including narrow sidewalks, the poor quality of sidewalks, the crowding of people, and vehicles parked on the sidewalks, are negative aspects that vary according to the gender of the interviewees. These aspects also cause negative emotions such as tiredness, anger, disgust, discomfort, and insecurity, especially in inner streets with low connectivity values. Residents of the Barrios Bajos neighbourhood note cars parked on sidewalks, the poor quality of sidewalks, and garbage on the streets as the most important barriers not only inhibiting walking, but also as features that promote negative emotions mainly related to anger, disgust, discomfort, and insecurity (Figure 9b). In addition, gender differences are highlighted in terms of the perceptions of elements that determine the neighbourhood’s walkability, especially regarding the social aspects of neighbourhoods rather than the physical ones, such as the crowding of people, and noise from vehicular traffic (especially by women residents), which are associated with a greater perception of insecurity. This coincides with recent international evidence found in other cities in Latin America [4,78,79,80] and differs with other recent evidence found in some cities in Europe and Asia [75,76].

## 5. Conclusions

This article aimed to evaluate the effects of the built environment on walkability in the central neighbourhood of Barrios Bajos in the city of Valdivia, Chile, based on walkers’ perceptions. For intermediate cities in the south of Chile that have undergone significant changes due to rapid expansion, it is crucial to encourage walking through urban design. Through the development of this study, it was demonstrated that the morphological qualities that the neighbourhood possesses as a system of interconnected streets, as well as its intermediate level of connectivity, are factors that promote a greater possibility of pedestrian movement.

Moreover, it was observed that certain services and facilities within the neighbourhood, such as the two health centres and Pastene Square, as well as the quality of the public space, such as the presence of trees and diversity of vegetation, good quality of sidewalks, and the facades’ characteristics and sense of belonging of architectural heritage encourage walking and the positive emotions of walkers. Furthermore, it is evident that the neighbourhood has elements that inhibit walking, such as narrow sidewalks, the poor quality of sidewalks, the presence of garbage, the crowding of people, and the vehicles parked on the sidewalks. This causes negative emotions in pedestrians, such as tiredness, anger, disgust, discomfort, and insecurity, with differences in gender and age of residents.

Based on the above, it is necessary to improve conditions to enhance walkability and encourage walking in these contexts through three main recommendations. First, the quality of the pedestrian flow paths should be increased by promoting the sections with greater connectivity and where movement patterns are defined. Second, the urban environment should be provided with a better spatial quality, related to increasing diversity of greening in some inner streets and improving building facades, especially those buildings with architectural value, to improve the urban image of the neighbourhood, which would increase tranquillity, happiness, and comfort in pedestrian activities. Third, the street design of General Lagos and Perez Rosales streets should be enhanced to have an inclusive street design that prioritises wide sidewalks to avoid crowding and includes proper parking places to increase the feeling of safety in pedestrian activities.

As the results of this article focused on a certain neighborhood case, caution should be exercised with generalization of results. The exploratory results of this study provided insights about which elements of built environment affect walkability in a central neighborhood in a city of southern Chile. Although the results given by the case of Barrios Bajos provided certain physical aspects to take into consideration, such us sidewalk width, building facades, public green areas, and social aspects such as the crowding of people and noise from vehicular traffic, more research is needed to compare these results not only with other cities in the country, but also with the region. In this sense, the representativeness of gender and age are aspects that need more attention for future studies in this region. In future studies, the use of visualization techniques such as virtual reality technology as new innovative methods, could be complementarily used not only to deepen these aspects but also to explore future scenarios for improving the urban design of neighborhoods.

Finally, urban design recommendations oriented to improve the quality and width of sidewalks, create building facades with characteristics of architectural value, design proper parking places on streets, and incorporate green infrastructure in inner city streets, appear to be priorities for promoting walking in this type of context. Redefining standards for sidewalk width (at the ordinance level) and increasing green areas with diversity of vegetation in streets and public spaces should be priority objectives for public investment in municipalities to promote a higher degree of pedestrianism in public spaces in southern Chile, and thus promote a better quality of life and well-being in these cities. This would allow for the reaching of a new definition of distance as a safe measure for any social interaction or pedestrian movement, and an urban environment with better spatial quality will increase comfort, tranquillity, and the feeling of safety in pedestrian activities.

## Figures and Tables

**Table 1:** Methodological structure.

Specific Objectives	Activities	Method
Measure and assess the levels of connectivity and diversity of land uses.	Measure the density of street centre lines and the number of intersections per unit of area. To evaluate diversity of land uses at street level.	- Axial analysis using Depthmap software [21]. - GIS - Simpson’s diversity index [90].
Assess the distance a person walks between their points of interest and the choice of routes.	Analysis of agents. Model the movement of people and how they use space.	People Following [20].
Analyse pedestrians’ perceptions about their mobility environment.	Application of walking interview in fieldwork to obtain records of self-declared perceptions of pedestrians about their urban mobility environment.	Walking interviews by Natural Go-Along method [22,23].

**Table 2:** Land use percentages and streets with a high diversity of land uses according to Simpson’s diversity index.

Street	Land Use (%)			Diversity Land Use
	Mixed Use	Residential	Non-Residential	Simpson’s Diversity Index
Lord Cochrane	2.21	57.95	39.84	0.66
General Lagos	1.21	56.09	42.7	0.69
Pérez Rosales (Pastene Square)	1.67	74.86	23.47	0.44
Baquedano	0.60	86.7	12.7	0.25
Aníbal Pinto	0	89.06	10.94	0.21
Domeyko	6.61	85.84	7.56	0.26
Santiago Bueras	1.88	90.56	7.56	0.18

## Data Availability

Not applicable.
